# Long Non-Coding RNAs of Plants in Response to Abiotic Stresses and Their Regulating Roles in Promoting Environmental Adaption

**DOI:** 10.3390/cells12050729

**Published:** 2023-02-24

**Authors:** Hong Yang, Yuting Cui, Yanrong Feng, Yong Hu, Li Liu, Liu Duan

**Affiliations:** State Key Laboratory of Biocatalysis and Enzyme Engineering, Hubei Collaborative Innovation Center for Green Transformation of Bio-Resources, Hubei Key Laboratory of Industrial Biotechnology, School of Life Sciences, Hubei University, Wuhan 430062, China

**Keywords:** lncRNA, evolution, abiotic stress, drought, heat, cold, salt, heavy metal, stress memory

## Abstract

Abiotic stresses triggered by climate change and human activity cause substantial agricultural and environmental problems which hamper plant growth. Plants have evolved sophisticated mechanisms in response to abiotic stresses, such as stress perception, epigenetic modification, and regulation of transcription and translation. Over the past decade, a large body of literature has revealed the various regulatory roles of long non-coding RNAs (lncRNAs) in the plant response to abiotic stresses and their irreplaceable functions in environmental adaptation. LncRNAs are recognized as a class of ncRNAs that are longer than 200 nucleotides, influencing a variety of biological processes. In this review, we mainly focused on the recent progress of plant lncRNAs, outlining their features, evolution, and functions of plant lncRNAs in response to drought, low or high temperature, salt, and heavy metal stress. The approaches to characterize the function of lncRNAs and the mechanisms of how they regulate plant responses to abiotic stresses were further reviewed. Moreover, we discuss the accumulating discoveries regarding the biological functions of lncRNAs on plant stress memory as well. The present review provides updated information and directions for us to characterize the potential functions of lncRNAs in abiotic stresses in the future.

## 1. Introduction

Genomic DNA serves as the template for the transcription of RNAs. However, it does not always serve as the template for protein synthesis. Although up to 90% of the genome can be transcribed into RNAs, only a small fraction of them turned into protein-coding messenger RNAs (mRNAs). Thus, non-coding RNAs were initially considered as “junk DNA” or “dark matter” [1]. With the advent of rapid development of genome-wide RNA detection techniques, such as microarray and high-throughput transcriptome sequencing, numerous evidence showed that non-coding RNAs have diverse roles in biological functions in organisms including prokaryotes, eukaryotes, and viruses [2,3]. Non-coding RNAs include housekeeping RNAs (tRNA and rRNA) and regulatory RNAs, which consist of small non-coding RNAs (such as miRNA and siRNA) and long non-coding RNAs (also termed lncRNAs). LncRNAs are widely accepted as transcripts that are longer than 200 nucleotides that do not code for proteins. In the early stages, the classifications, nomenclature, and terminology of lncRNAs were confusing, due to their low conservation and differences in functionalities compared with the coding genes [4]. Based on empirical attributes, lncRNAs could be classified by associations with annotated protein-coding genes or DNA elements of known function, by their sequence or structure conservation, by biological or biochemical pathways, and by their functions, etc. [4]. However, these descriptive and distinctive properties could only capture a very small fraction of lncRNAs, and are not mutually exclusive with unavoidable shortcomings in comprehensive assessment. Although lncRNAs were primarily recognized as non-coding transcripts, some studies in the past years have found that lncRNAs could encode small polypeptides (small open reading frames containing fewer than 100 codons, smORFs) in various species [5]. Furthermore, some lncRNAs might have dual functions as both micropeptides and regulatory RNAs, such as ENOD40 [6], which expanded the complexity and definition of lncRNAs. With new findings in the future revealing the real dimensions and complexity of plant non-coding transcriptome, the definition and classification of lncRNAs might continue to evolve [7,8].

In recent years, lncRNAs have been evidenced to play diverse roles in plant growth and development, interacting with other biomolecules, especially pathogens, and modulating environmental biotic and abiotic stress responsiveness [9,10,11]. However, compared with mammals, the annotated functional lncRNAs in plants are still limited due to the ambiguity and versatility of lncRNAs. Therefore, our knowledge of plant lncRNAs is just the beginning and remains under-explored. The goal of this review is to explore the roles of lncRNA in plant adaptation to abiotic stresses encountered in the environment, from a new perspective. At the beginning of this review, we will emphasize the criteria and features of lncRNA, discuss the evolution of lncRNA in land plants, and summarize the growing literature on the roles of lncRNA that take in plant stress responses including heat, cold, drought, salt, and heavy metal stresses. Next, the roles of lncRNAs that might contribute to plant stress memory will be reviewed as well. Finally, we will outline the unsolved problems in the field and propose a roadmap for future directions and opportunities.

## 2. Features of LncRNA in Plants

Like mRNAs, lncRNAs are transcribed by nuclear RNA polymerases in plants and undergo similar post-transcriptional modifications. All five RNA polymerases in plants, including Pol I, II, III, and two Pol II-related, plant-specific RNA polymerases (Pol IV and Pol V) have been observed to transcribe their diverse lncRNA products, involving RNA-directed DNA methylation and regulating transposable elements in plants [8,12]. After their transcription, lncRNAs are subject to RNA capping, splicing, polyadenylation, and nuclear export to assure their proper structure, localization, and function [13]. Therefore, lncRNAs are composed of 5’ and 3’ untranslated regions (UTR), introns, and exons, such as protein-coding genes. However, the abundance and efficiency of polyadenylation of lncRNAs are lower than mRNAs in general [14]. Nonetheless, lncRNAs could generate multiple splice variants from one gene and display a remarkable degree of alternative splicing (AS). AS of lncRNAs have been explored and analyzed by ultra-deep RNA-seq analysis of a diel time-series in response to cold treatment, which showed dynamic expression and AS of lncRNAs [15].

Although lncRNAs contain fewer numbers of exons [16] and are expressed at lower levels than mRNAs [17], they were also found to be expressed in tissue- and cell-type-specific ways in many plant species, such as rice [18] and grapevine [19]. In grapevine, differentially expressed lncRNAs in leaf, inflorescence, and berry were discovered, revealing spatiotemporal and developmental stage-specific regulation of lncRNAs [19]. Spatial-temporal expression of lncRNAs was also found in maize, and over 90% of them were expressed specifically in a certain tissue or at a certain development stage [20]. The cell-specific expression profiling results from Arabidopsis root showed that only 25% of intergenic lncRNAs (lincRNAs) were expressed in more than half of the cell types [21].

Localization of lncRNAs in the cells could provide important clues to their functions. Most lncRNAs were found to be preferentially localized in the nucleus, linking to their functions of chromatin organization and regulating gene transcription [22,23]. RNA fluorescence in situ hybridization (FISH) and lncRNA promoter-driving reporter techniques could be employed to determine the subcellular localization of newly identified lncRNAs. Until now, some lncRNAs have also been found cytoplasmically located in *Drosophila* and mammals such as humans and mice [22,23]. However, studies with solid evidence in plants are still limited.

**Figure 1:**
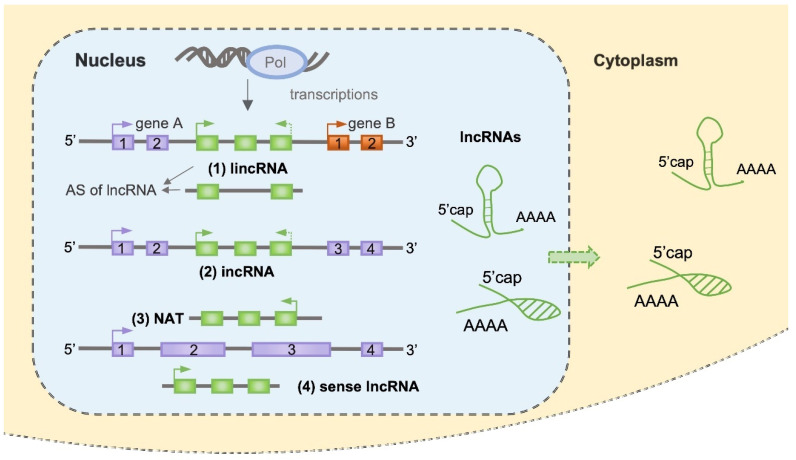
Classification of lncRNAs based on their orientation and positions related to other genes. LncRNAs are transcribed by nuclear RNA polymerases in plants. Based on their orientation and positions related to other genes, the major classes of lncRNAs could be classified as (1) lincRNAs (found in intergenic regions), which could be transcribed in both directions; (2) incRNAs (intronic ncRNAs), which could be transcribed in both directions; (3) NATs (natural antisense transcripts occur in most protein coding genes); (4) sense lncRNAs, overlapping the protein coding genes. LncRNAs could generate multiple splice variants from one gene and display a remarkable degree of AS (alternative splicing), which showed up in lincRNA as a representative. Cytoplasmic LncRNAs have been found in Drosophila and mammals, while there is still a lack of solid evidence in plants.

The distribution of lncRNAs in the genomes of different plant species showed diverse patterns. Uneven chromosomal distribution of lncRNAs was found in grapevine [19], while, on the other hand, they are evenly distributed in the maize genome with only a slightly lower density in chromosome 1 [20]. Based on their orientation (sense or antisense) and positions related to other genes (intergenic, intronic, and coding areas), the major classes of lncRNAs could be classified as (1) lincRNAs (found in intergenic regions); (2) intronic ncRNAs (incRNAs); (3) natural antisense transcripts (NATs, occur in most protein coding genes); (4) sense lncRNAs (Figure 1). LncRNAs could also be classified according to their biochemical pathways, sequence or structural conservation, functions, and genomic locations [4].

## 3. Evolution of LncRNA in Plants

Evolutionary conservation has been used as an indicator of genes, and homology search using nucleotides or amino acid sequences is an applicable method for protein-coding genes. However, for lncRNAs, the criteria are considered to be too narrow for their less conserved sequence level compared to coding genes, but more conserved than random intergenic regions or introns. Four dimensions of lncRNA conservation including sequence, structure, function, and syntenic transcription at a given genomic locus were proposed based on the findings in human lncRNAs in 2014 [24]. Strategies such as whole-genome alignment of the lncRNA sequence, direct comparison with lncRNA sequences in other species, structure or profile comparison, and genome position of orthologues of the neighborhood could be used to identify the homologs of a lncRNA of interest in different plant species [25,26,27]. Although the understanding of plant lncRNAs remains limited, one good example is lncRNA *COOLAIR*, which provides evidence that the structure and function of lncRNAs remain conserved in plants [28,29,30]. Genome-wide annotation and analysis showed that 37% of high-confidence lncRNAs are conserved between maize and teosinte [30]. Unlike animals, terrestrial plants have evolved a more complicated mechanism of transcription by expanding the RNA polymerase family with two Pol II-related enzymes [31], thus establishing novel regulation mechanisms of lncRNAs in plants adapting to their land life.

In addition, not only does the nucleus encode lncRNAs, but also mitochondria-encoded lncRNA (mt-lncRNA) have been identified and functionally characterized in humans in 2007 [32,33]. Both mitochondria and chloroplasts are semi-autonomous organelles, which evolved from free-living prokaryotic organisms through endosymbiosis to organelles in eukaryotes. Although the information about mitochondria-encoded and chloroplasts-encoded lncRNAs in plants is very limited, reports show that ncRNAs are conserved across cyanobacteria [34]. In addition, hundreds of ncRNAs were found encoded by chloroplasts of Arabidopsis [35], meaning they should not be overlooked for their potential as players in gene regulation in land plant adaption to environmental stresses [36,37].

Large-scale comparative analysis of lncRNAs in different species has become a powerful tool for studying the functions of lncRNAs [25,38,39,40,41,42]. It requires two main ingredients for the comparative analysis of lncRNAs: (1) genomes and datasets of lncRNAs that can be compared, and (2) algorithms to identify and evaluate lncRNAs. The studies of mammalian lncRNAs showed that compared to the neutral expectation, lncRNAs had a reduced incidence of mutations in their promoters and exons, and increased conservation of splice sites under negative evolutionary constraint [38,43]. Splicing patterns of lncRNAs in mammals were also found to evolve rapidly [25,39,40]. On the other hand, studies in plants have shown that lncRNA exons evolve faster than protein-coding genes, by comparing the turnover of the DNA sequences and assessing the degree of the contribution of the primary sequences [25]. Higher conservation in lncRNA exons was observed compared with introns or random intergenic sequences within the lncRNA loci in rice [18]. The splice rates of lncRNAs were much lower in rice [18] and Arabidopsis [44] than in humans [45].

**Figure 2:**
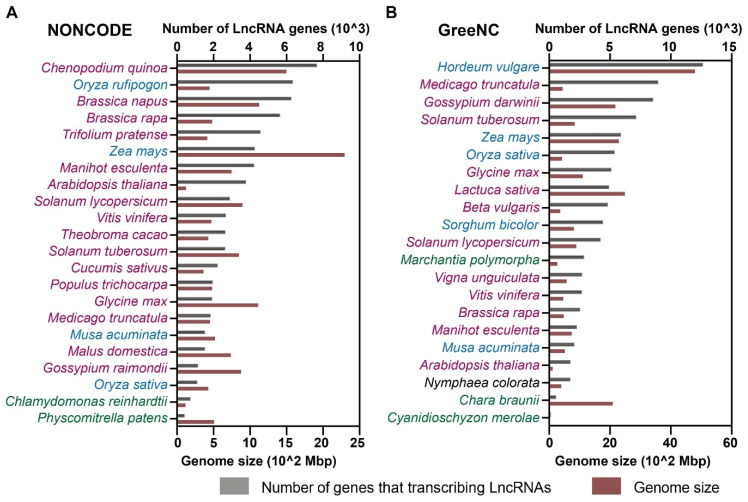
Number of genes transcribing lncRNAs and the genome size of plants. (**A**) LncRNA information extracted from NONCODE. (**B**) LncRNA information extracted from GreeNC. The red color of characters represents plant species in Eudicotyledoneae; blue color represents plant species in Monocotyledoneae; black means plant species in basal Angiosperms; and green color means plant species in cladogram of non-flowering plants. The numbers of genes that transcribe lncRNAs are represented by gray boxes, and the genome sizes are represented by red boxes.

Recent studies in plants have begun promisingly to take the first steps for comparing and understanding plant lncRNAs from the perspective of evolution [26,27,46]; although, the species whose lncRNAs have been well annotated are still limited. The rapid development of high-throughput RNA sequencing technologies provides a broader range of lncRNA transcriptomes across plant species. In the meantime, filters and tools were developed and used to detect and identify candidate lncRNAs individually or in combination. The databases of plant lncRNAs and resources for discerning their functional properties have been well documented in recent reviews [47,48,49,50]. In this review, we summarized the most recent information in public databases including NONCODE v6.0, PNRD, AlnC, PLncDB v2.0, LncPheDB, CANTATAdb v2.0, and GreeNC v2.0 (Table 1) [42,51,52,53,54,55,56]. Among these databases, lncRNAs were predicted, annotated, or validated in over eight hundred plant species, including algae, bryophytes, pteridophytes, gymnosperms, and angiosperms. Detailed plant species that have been included in each database are listed in Supplemental Table S1. As mentioned previously, lncRNAs could generate multiple splice variants, thus some of the databases provided gene numbers of lncRNAs in addition to the lncRNA transcript numbers. In higher eukaryotes, increasing genome size usually correlates with lncRNA numbers in general [57,58]. To verify this theory in plants, the gene numbers that transcribe lncRNAs from the plant species in NONCODE and GreeNC were extracted and compared with their plant genome sizes (Figure 2). Although the numbers of lncRNAs are influenced by the depth of RNA-seq, the annotation of the plant species, and the filters they are using, the current finding gives us some interesting notions about their relations to genome size (Figure 2). It is worth noting that the correlation seems not to be high in plants; this may be due to the whole genome duplication and triplication events, and the polyploidization that happened during plant evolution. An example is that the number of lncRNAs in plant species in Poaceae, such as *Zea mays* and *Hordeum vulgare*, is not outnumbered by *Triticum aestivum*, despite the big difference in their genome sizes (17,000 Mbp in *Triticum aestivum*).

Primary sequence conservation analysis across 10 plant species revealed that lncRNAs are more conserved in the intra-species and sub-species than in the inter-species [59], which suggests that most lncRNAs evolved relatively recently [57]. Homologous lncRNA is merely detectable beyond 50 million years of species divergence in mammals [25]. However, in plants, 90 of a total of 5497 lncRNA families that have been identified were found conserved, and originated more than 180 million years ago. They have a fast evolutionary rate, and they tend to be more conserved between closely related species [46]. LncRNAs in thirty-five plant species were analyzed and compared to Chinese cabbage, including 18 eudicots, 14 monocots, 1 angiosperm, 1 fern, 1 moss, and 1 green alga. Their results showed that relatively high sequence similarity was detected in four Brassicaceae, but no homologous lncRNAs were detected in the moss *Physcomitrium patens* (*P. patens*) [17]. On the contrary, although the evolutionary conservation of lncRNAs is significantly lower than mRNAs at the nucleotide level, numerous lncRNA-smORFs were found to be conserved across 479 different plant lineages at the amino acid sequence level [16]. About 83% of highly conserved lncRNAs-smORFs were distributed only in moss species and the number of conserved smORFs rapidly dropped at the transition from mosses to higher plant lineages. The conclusion is that most smORFs located on lncRNAs are evolutionarily young [16]. Another large-scale evolutionary analysis of plant lncRNAs was conducted using the datasets of 25 species of flowering plants, including monocotyledons and dicotyledons [46]. LncRNAs in Arabidopsis were grouped into five categories based on the degree of their conservation, which were (1) conserved in Arabidopsis, (2) in Brassicaceae, (3) in dicotyledons, (4) in angiosperms, and (5) with no conservation. Among them, 84.4% of total lncRNAs were grouped into the no conservation group, while only 2.1% of the total account for dicotyledon and angiosperm conserved groups. Their results showed that the conserved lncRNAs in Arabidopsis have lower gene numbers, longer sequence length, more exons in the intron/exon structure, higher expression levels, and a lower proportion of tissue-specific expression compared to the non-conserved group. In contrast, the genomic location of lncRNAs in each category was similar [46].

Based on the results of GO analysis, which showed that conserved lncRNAs were enriched in leaf response to stimulus, stress, cell death, and signal transduction [46], conserved lncRNAs may be subjected to greater selection pressure during evolution while adapting to the abiotic stresses. On the other hand, two *Eutrema salsugineum* ecotypes were compared, one from the semi-arid subarctic Yukon of Canada and one from the semi-tropical monsoonal of China, and only a negligible overlap was found between the two ecotypes [60]. Therefore, different environments could lead to the local adaptation of plants and result in differences in lncRNA expression under the same stress condition, contributing to the fast evolution of lncRNAs.

## 4. Abiotic Functions of LncRNA in Plants Adaption to Environment

### 4.1. LncRNAs Respond to Abiotic Stresses, Including Drought, Temperature Fluctuations, Salinity, and Heavy Metal Stress

The algal lineage began terraforming the terrestrial habitat more than half a billion years ago, enabling green life to live in very diverse habitats by overcoming abiotic challenges, such as drought stress, temperature fluctuations, salinity stress, and heavy metal stress [61]. Hundreds of studies have shown that plant lncRNAs could be induced by various abiotic stresses.

**Figure 3:**
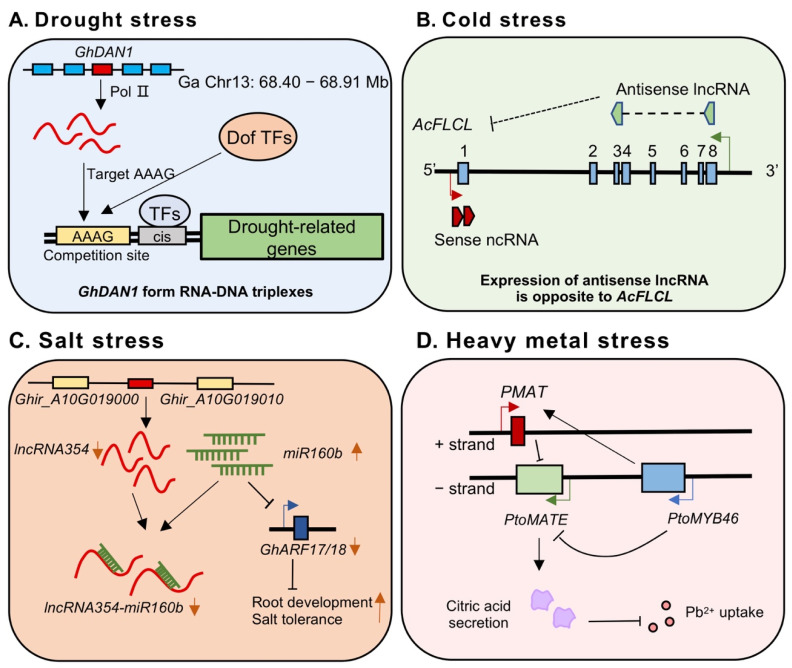
Representative examples of how plant lncRNAs respond to various abiotic stresses. (**A**) *GhDAN1* in cotton, which is probably transcribed by Pol II, binds to an AAAG-rich DNA motif (corresponding to the core binding site of Dof TFs), forming the RNA-DNA triple. Drought stress reduced *GhDAN1* expression, thus releasing the AAAG motif site of the drought-related genes to adapt to environmental drought stress. (**B**) An antisense long non-coding RNA confined to the distal end of the *AcFLCL* locus was found in kiwifruit, showing an opposite expression pattern to *AcFLCL* under cold stress. The black dashed line indicates that the regulatory pathway requires further verification. (**C**) The expression of *LncRNA354* in upland cotton, which is located between *Ghir_A10G019000* and *Ghir_A10G019010*, is decreased under salt stress, thus impairing the binding to *miR160b*. Therefore, up-regulating the accumulation of mir160b, and inhibiting the expression level of *GhARF17/18* to enhance salt stress tolerance. The arrows in orange represent the expression changes. (**D**) In poplar, LncRNA *PMAT* interacts with *PtoMYB46* to inhibit the expression of *PtoMATE* under Pb^2+^ stress. The downregulation of *PtoMATE* decreased the secretion of citric acid and increased the absorption of Pb^2+^. The black arrows denote positive effects, whereas lines ending with a short bar indicate negative effects.

Water plays a crucial role in plant life. Drought (including dehydration and desiccation in this review) is a major stressor during the plant landing process. Therefore, drought stress can affect plant development, yield, and survival, and results in ecological, morphological, physiological, and biochemical changes in plants. Recent findings identified several lncRNAs that respond to drought stress, including in rice [62], maize [63], wheat [64], tomato [65], cassava [66], switchgrass [67], *Eutrema salsugineum* [60], rapeseed [68], *Oryza rufipogon* [69], *Populus trichocarpa* [70], *Brassica juncea* [71], *Cleistogenes songorica* [72], etc. Examples include that different DEGs of lncRNAs that respond to drought stress were found in varieties with contrasting drought tolerance in peanut [73]; *GhDNA1* was found to be associated with drought tolerance in cotton, which targets AAAG DNA double strands to regulate drought-responsive genes in *trans* (Figure 3A) [74]. These results support the conclusion that lncRNAs could be induced or suppressed in response to drought stress. Furthermore, these drought-responsive lncRNAs also have been reported to be associated with phytohormone signal transduction, biosynthesis of secondary metabolites, and sucrose metabolism pathways, which are related to the plant drought response [66,75,76,77].

Temperature fluctuations are another key factor limiting plant distribution, global crop yield, and production. Extreme climate changes have become more common as a result of global warming, causing significant environmental problems. LncRNAs have been found in the plant response to cold or heat stresses in Arabidopsis [78,79,80,81], Chinese cabbage [17], grapevine [82], banana [83], *Brassica juncea* [71], wheat [84], maize [85], cassava [86], alfalfa [87], etc. Natural long non-coding antisense heat-inducible *asHSFB2a* could regulate the expression of *HSFB2a*, one of the central regulators of the heat stress response in Arabidopsis [81]; in cotton, cold-responsive lncRNA *XH123* is actively involved in the tolerance of cold stress at the molecular level during the seedling stage [88]; *Cold induced lncRNA 1* (*CIL1*) in Arabidopsis was identified and experimentally proved to be a positive regulator in response to cold, by affecting hormone signal transduction, ROS homeostasis, and glucose metabolism [89]. Cold stress promotes the expression of *COOLAIR*, a set of alternatively processed antisense noncoding transcripts that reduce the expression of *FLC*, by recruiting protein complexes that impact chromatin states, or changing the abundance and shape of structural conformations [13,90]. A gene with homology to *FLC* was recently identified in kiwifruit, which also responds strongly to cold. The antisense lncRNA had an opposite expression pattern compared to *AcFLCL*, implying a model similar to Arabidopsis *COOLAIR* outside the Brassicaceae (Figure 3B) [91]. These results showed that lncRNAs are involved in plant responses to temperature stress.

Salt stress negatively affects plant growth and triggers adaptive selections of the natural habitats of the plants, causing a serious threat to the environment and affecting human health. Salt stress responsive lncRNAs were explored in Arabidopsis [76], sweet sorghum [92], barley [93], cotton [94], alfalfa [95], soybean [96], duckweed [97], chickpea [98], *Arachis hypogaea* L. [99], etc. It was found that the 154 and 137 lncRNAs were differentially expressed in the less salt-tolerant cultivated M82 tomato genotype and the highly salt-tolerant *S. pennellii* under salt stress, with 73% of DE-lncRNAs in *S.pennellii* being down-regulated and 86% of DE-lncRNAs in M82 being up-regulated [65]. Knockdown of salt-inducible *lncRNA973* in cotton showed reduced salt tolerance, and *lncRNA973* overexpression lines had increased salt tolerance [94]. On the contrary, the expression of *lncRNA354* was found to be decreased under salt stress, which weakened the binding to *miR160b*, a suppressor of *GhARF17/18*, thus up-regulating the amount of *mir160b* and enhancing root development, thereby synergistically regulating cotton salt stress tolerance (Figure 3C) [100]. The evidence suggests that lncRNAs are involved in the response to salt stress.

Heavy metal pollution is becoming a serious environmental problem around the world. High levels of heavy metals in soil can harm plant development and survival, severely limiting plant growth, agriculture, and forestry. Moreover, heavy metals may eventually enter the food chain, with serious consequences for human health. In recent years, plant lncRNAs have been found to respond to heavy metals such as lead (Pb) [2], iron (Fe) [101], copper (Cu) [102], manganese (Mn) [103], and aluminum (Al) [104]; however, the mechanisms were less studied. In Poplar, the antisense lncRNA *PMAT* (Pb^2+^-induced multidrug and toxic compound extrusion, *MATE*) was reported to epistatically interact with *PtoMYB46*, and *PtoMYB46* depresses the expression of *PtoMATE* directly or indirectly through *PMAT*, thereby reducing the secretion of citric acid (CA) and ultimately promoting Pb^2+^ uptake. Therefore, a *PtoMYB46*–*PMAT*–*MATE* pathway has been proposed to positively modulate Pb^2+^ uptake [2] (Figure 3D). These results suggest that lncRNAs are good indicators of heavy metal stresses and could be important regulators of plant response to heavy metal stresses.

### 4.2. Methods to Identify LncRNAs and Characterize Their Functions in Abiotic Stress

With the advantage of high-throughput RNA sequencing technologies, plant lncRNAs can be identified and annotated on a genome-wide scale. However, detection of lncRNAs usually requires a specific enrichment strategy at the library preparation stage due to their low expression levels. In general, paired-end sequencing is preferred over single-end. To identify lncRNAs, brief but practical guidelines are provided. Firstly, RNA-seq reads were mapped using either a reference-guided or de novo approach to reconstruct transcript models. Secondly, non-coding transcripts were set apart from the coding transcripts with information on the exon-intron structure. Lastly, assembled transcripts are filtered by transcript length, protein-coding potential, and characteristics similar to mRNA to identify lncRNA-producing loci. Transcripts could be extracted with the filter of >200 nt based on their size. Alignment-based methods using BLASTx with models from protein databases (such as NCBI refseq and Ensemble), or programs that discover possible protein-coding transcripts (such as PhyloCSF and RNAcode [105,106]) could be used to remove overlapping protein-coding genes. In addition, several alignment-free computational tools have been developed to assess the coding potential, such as CPC [107], LncFinder [108], lncScore [109], COME [110], and PLIT [111], to eliminate protein-coding RNAs. Detailed bioinformatic tools for lncRNA prediction and analysis have been well reviewed and documented previously [48,50,112], with more and more plant lncRNA identification and prediction tools, packages, algorithms, and pipelines been explored recently, relying on motifs, structures, homologs, feature relationships, and interactions of plant lncRNAs. Examples are sORFPred [113], DeepPlnc [114], LGC [115], PRPI-SC [116], PINC [117], LncMachine [118], etc. Although lncRNAs in different plant species have been explored and shared in the databases mentioned previously, the numbers and coverage of lncRNA loci in each study were largely associated with transcriptional complexity and the criteria that were used. Therefore, after the successful mining of plant lncRNAs, the candidates need to be experimentally validated, such as by mass spectrometry and ribosome profiling. Experimental validation could further improve the detection power of prediction tools.

Despite a broad range of estimates for the numbers of lncRNAs in plants, the functional activity of most lncRNAs is still uncovered. In addition, not all lncRNAs that are expressed differently in response to various stress conditions have been functionally or experimentally validated. Only 506 lncRNAs in 57 plant species were listed with confirmed functions in EVLncRNAs v2.0, a database that provides information from low-throughput experiments (such as qRT-PCR, knockdown, Northern blot, and luciferase reporter assays). In recent years, accumulating evidence has supported the cellular functions of lncRNAs that were involved in plant response to abiotic stresses, with most of them confirmed by qRT-PCR. Representative lncRNAs were listed in Table 2. EVlncRNA-Dpred, which was developed using deep learning algorithms to separate low-throughput experiments from high-throughput sequencing RNAs and mRNAs [119], would be a useful tool for screening lncRNA transcripts for experimental validation. More computational strategies, methods, and toolboxes need to be developed and applied to discover plant lncRNAs based on their unique and similar characteristics in the future.

Most lncRNAs reported act as promoters and enhancers, which correspond to the fact that most of them are localized to the nucleus [153]. This indicated that the roles of lncRNAs are more likely to be regulators and to function in *cis* or *trans* than the lncRNA itself. However, it is important to know if the lncRNA has the ability to create small peptides by ribosome footprints [154]. Exon skipping strategies could be checked for the function of the micropeptides to remove the ORF from the main lncRNA.

To identify the expression patterns of lncRNAs under abiotic stresses and characterize their exact biological roles, the following methods could be used to verify their functions. Once a researcher discovers a lncRNA locus, the rapid amplification of cDNA ends (RACE) and reverse-transcriptase (RT)-PCR could be used to define the 5’- and 3’-ends of the transcript, as well as the multiple variants that may be caused by alternative splicing. The expression pattern of the lncRNA under different abiotic stress conditions and the tissue-specific expression pattern throughout the lifetime of plants could be confirmed by real-time qRT-PCR. Subcellular localization, GUS staining, and RNA FISH reveal the localization and quantitative information of lncRNAs [155]. High-throughput techniques such as cap analysis of gene expression (CAGE), polyA site sequencing, and RNA-seq could characterize the structure of lncRNAs as well.

Expression profiling and co-expression network analysis of lncRNAs in multiple sample types and under various abiotic conditions are the most routine and reliable methods to reveal the possible biological processes in which they might function. Enrichment of specific biological functions or pathways of the co-expressed transcripts could be informative to annotate the lncRNAs. The regulatory network upstream of lncRNAs could be confirmed by ChIP-seq to identify all abiotic stress-related transcription factors that bind to the sites upstream or within the lncRNA. Loss- and gain-of-function experiments are important steps to understand the function of lncRNA and determine whether it acts locally in *cis* or whether it leaves the site of transcription and acts in *trans*. Mutant lines of the lncRNA synthesis locus, either mutated in the lncRNA promoter or the entire lncRNA transcript body, could be searched and purchased from the public library if available. Their functions could also be analyzed using CRISPR, RNAi, and overexpression lines of lncRNAs, which are generated by genetic transformation depending on the plant species. Transcriptional abundance changes of lncRNA could be confirmed by comparing it to its wild-type using qRT-PCR. The genotype, phenotype, and physiological changes of lncRNA modification lines during development and under various abiotic stresses need to be checked no matter whether lncRNA acts in *cis* or *trans* mode.

Cis-acting lncRNAs directly act on one or several linked genes on the same chromosome, which are restricted to the site of the lncRNA’s synthesis. Altered expression of nearby genes suggests the cis-acting function of lncRNA. The cis-action function could be further confirmed by the complementary experiment using knock-in or heterologous expression of the lncRNA. To distinguish the function of the lncRNA from the function of the DNA regulatory elements embedded in the lncRNA locus, a poly A signal and its mutated control could be inserted near the 5’-end of the lncRNA to terminate transcription while not affecting the function of the DNA elements. Trans-acting RNAs diffuse from the site of synthesis and act at great distances on many genes, even those located on other chromosomes. If the expression level of nearby genes does not change, the lncRNA is more likely to act in a *trans* function, which could also be confirmed by a complementary experiment using heterologous expression of the lncRNA in mutant lines.

To identify the potential interacting partners of lncRNAs, co-expression and network analysis provide the first clues to reveal the same biological function. Nucleic acids and proteins that interacted with lncRNAs could be identified using RNA immunoprecipitation assays (RIP), biotinylated RNA pull-down assays, or through combination with high-throughput strategies, such as RIP-seq. In-depth interactions of lncRNAs with DNA/chromatin, RNA, or proteins could be identified using all-to-all or one-to-all strategies [14]. Evolutionary sequence analyses of conserved primary sequences, 2D, and 3D structures could be used to identify potential functional binding elements within lncRNAs. Measurements of phytohormones, proline content, ROS levels, and other stress-related biological and physical indices are useful in identifying the downstream signaling pathways affected by the lncRNA and in clarifying the roles of the lncRNA in response to abiotic stresses. Detailed protocols for the approaches mentioned above have been well reviewed and documented in previous reviews [153,156,157,158].

### 4.3. Mechanisms of LncRNAs in Regulating the Responses of Plants to Abiotic Stresses

To date, plant lncRNAs have been proven to be involved with abiotic stresses experimentally, acting as positive or negative regulators [17,159,160,161]. Plant lncRNAs have also been found to affect hormone signal transduction, ROS homeostasis, and carbohydrate metabolism [17,65,89,162]. A major mechanism of lncRNA function is transcriptional level regulation, via interaction with DNA or regulation of transcription factors. Elements of genes could be affected by lncRNAs, including promoters, exons, introns, untranslated regions, and terminators. LncRNA *DglncTCP1,* which is transcribed from the antisense strand of the transcription factor *TCP1* in chrysanthemum, activates the expression of *TCP1* and plays a cis-regulatory role to regulate cold tolerance responses [163]. In addition, it could bind to the same target of transcription factors and co-regulate or deregulate the target gene promoter, such as APOLO/WRKY42. Thus, it could regulate downstream genes for enhancement of root hair growth under low temperature and cold stresses in Arabidopsis [79,80]. Nuclear lncRNAs have also been reported to directly bind to and activate or repress transcription factors under abiotic stress as signal molecules [82,161]. Other than transcription factors, lncRNAs were found to interact with splicing factors to condition their stability and subcellular localization [158,164]. In Arabidopsis, the lncRNA *ALTERNATIVE SPLICING COMPETITOR (ASCO)* could hijack nuclear AS regulators to modulate alternative splicing patterns in response to auxin [165]. Further research also indicated that *ASCO* could integrate a dynamic network, including spliceosome proteins, to modulate transcriptome reprogramming [166].

Furthermore, lncRNAs could serve as structural or regulatory molecules to affect gene expression through the interaction of RNA under abiotic stresses. LncRNAs were found to function as competing endogenous RNAs (ceRNAs), stabilizing the mRNA and regulating rice responses to drought stress [62]. LncRNAs could also act as ceRNAs or endogenous target mimic (eTM) to competitively regulate the target genes of the miRNA. Twenty lncRNAs were reported as target mimics of the known miRNAs in Populus in response to drought stress [70]. Two pairs of miRNAs and lncRNA interacting partners were discovered in soybean in response to salinity stress [144]. Some lncRNAs could also act as precursors of miRNAs [82]. Large-scale analysis revealed that the miRNA precursors derived from lncRNAs were species-specific. The average percentage of lncRNAs acting as miRNA precursors was 0.50% among 37 plant species, with the highest in *P. patens* (3.27%) [17]. On the other hand, lncRNAs could also act as the target of miRNAs to produce phased small interfering RNAs (phasiRNAs) for the regulation of plant abiotic responses [159,167].

Recently, numerous smORFs have been discovered embedded in lncRNAs in different organisms, including plants, mammals, fungi, and bacteria [8,168]. LncRNA-smORFs in moss plant *P. patens* were analyzed comprehensively and systematically across 479 plant species, with numerous smORFs validated and functionally characterized experimentally [16]. Some of the lncRNAs-smORFs contain signal peptides and transmembrane domains with lower GC content and are located in AU-rich regions, which suggests their roles in cell-to-cell communication. Furthermore, they found that ~10% of the confirmed translation smORFs were hydroxyproline or proline-rich peptides. These smORFs could regulate photosynthesis, the generation of precursor metabolites and energy, and oxidoreductase activity, suggesting the roles they may play in plant development and abiotic stress tolerance [16].

Epigenetic modifications of genomic DNA and histones could influence gene expression. Genome regulation via DNA methylation and post-translational histone modifications, called RNA-mediated transcriptional gene silencing (TGS), is a common function of plant lncRNAs in response to abiotic stresses. In plants, lncRNAs can be produced by two specialized RNA polymerases, Pol IV and Pol V, controlling DNA methylation, which are essential for RNA-directed DNA methylation (RdDM). Plant RdDM relies on Dicer-like 3 and AGO4, which produce small interfering RNAs (siRNAs) from long double-stranded RNA and bind siRNAs to function, respectively. The 24 nt siRNAs are cleaved from lncRNAs by DCL3, and are methylated and bind to AGO to form the AGO–siRNA complex. LncRNAs act as scaffold RNAs that are recognized by the siRNA–AGO complex through sequence complementarity to form AGO4–siRNA–lncRNA, and then target the chromatin [96,169,170,171]. DNA methylation is catalyzed by DNA methyltransferases and plays a key role in plant abiotic stress responsiveness [172]. LncRNAs have been shown to regulate target genes under abiotic stress conditions by recruiting DNA methyltransferases or demethylases, regulating their DNA methylation [96,173,174,175].

Nuclear DNA is wrapped around histone octamers in nucleosomes, and gene expression is closely associated with chromatin topology. Transcriptional regulation was also affected by histone modifications such as methylation, acetylation, phosphorylation, and ubiquitination under various abiotic stresses. LncRNAs could guide RNA–protein complexes to bind to specific locations and interact with chromatin-modifying enzymes to target genes [140,176,177]. In addition, lncRNAs were found to regulate gene expression by mediating changes in chromatin structure, such as nucleosome positioning, chromatin remodeling, and chromosome looping. For example, lncRNAs transcribed by Pol V could interact with and serve as a binding scaffold for Involved in de novo 2 (*IDN2*), an RNA-binding protein that is required for RdDM and physically interacts with a subunit of the SWI/SNF complex (ATP-dependent chromatin remodeler), stabilizing the specific nucleosomes [178]. In Arabidopsis, the *AUXIN-REGULATED PROMOTER LOOP* (*APOLO*) locus, which is transcribed by Pol V and recognizes its targets by short sequence complementarity and the formation of R-loops, is involved in modulating [179,180].

Therefore, lncRNAs can function as a guide, a nucleator, a scaffold for numerous complexes, a template, a decoy, or a signal. The mechanisms by which lncRNAs epigenetically regulate gene expression have been well reviewed [181], but how they are involved in important cellular processes in plants under abiotic stresses needs to be further investigated in the future.

## 5. The Roles of LncRNAs in Plant Stress Memory

Plants have evolved sophisticated regulatory systems to adapt to diverse abiotic stress challenges, especially those that constantly happen, such as drought and extreme temperatures. Some plants could develop stress responses after the first stress stimuli, which leads to enhanced tolerance or resistance the next time the stress is encountered, which is called stress memory [182]. So far, Arabidopsis [183,184], maize [185,186], rice [123], *Boea hygrometrica* [187], and switchgrass [67] have been proven to establish drought stress memory after repeated exposure to drought or dehydration. A total of 238 lncRNAs involved in drought memory responses were found in rice and showed markedly different expression levels in subsequent drought treatments than in the first drought stress [123]. Association analysis of lncRNAs and mRNAs also showed that some memory-related mRNA transcripts, including serine/threonine-protein kinase, and phenylalanine ammonia-lyase, were associated with lncRNAs [123]. Furthermore, 12 drought memory miRNAs were generated from lncRNAs in rice [123]. The molecular responses to multiple dehydration stresses of lncRNAs in switchgrass, which is an excellent biofuel feedstock and soil-conserving plant, were researched systematically. A total of 441 differentially expressed lncRNAs were found during multiple dehydration stresses, and among them, 39 lncRNAs were annotated and suggested to play important roles in dehydration stress memory [67]. GO and KEGG analysis of the antisense genes, upstream, and downstream genes of the lncRNAs showed enrichment in the GO term of “response to stress”, and pathways of “biosynthesis of amino acids”, “plant hormone signal transduction”, “aminoacyl-tRNA biosynthesis”, “ribosome”, “phenylpropanoid biosynthesis”, “starch and sucrose metabolism”, and “glycolysis” [67].

On the other hand, recent reports have shown that the establishment and maintenance of plant short-term and long-term stress memories are associated with and governed by epigenetic processes and chromatin dynamics, including DNA methylation, histone modifications, and RNA-directing modifications [172,187,188,189,190,191]. LncRNAs are widely involved in regulating plant epigenetic modification, plant hormone (such as ABA and ethylene) biosynthesis and signal transduction, and alternative splicing [67,123,192]. These imply that lncRNAs may play roles at sophisticated levels in plant stress memory, and functional studies of lncRNAs in plant stress memory are still very limited and need to be investigated further.

## 6. Future Directions and Conclusions

As a very challenging group of transcripts to study, the diversity of lncRNA molecules, sequences, and structures is growing rapidly, and their functional mechanisms are broad topics. We have tended to focus on how lncRNAs are conservatively evolving in plants to adapt to the land environment, as well as their roles in plant response to abiotic stresses and the establishment of stress memories.

Increasing pieces of evidence provided in different plant species prove that lncRNAs have critical roles in abiotic stress responses. However, most functional plant lncRNAs were found in angiosperms, while fewer lncRNAs were tested in bryophytes and ferns. Studies of lncRNAs in a wider range of plant species will help understand the evolution and diversity of their functions in adapting to different environments. On the other hand, most studies on stress-responsive lncRNAs have been focused on a single stress type or single stress, with only a few studies considering the roles of lncRNAs under multiple stresses in combination or under repeated stress exposures. Therefore, more effort is needed to discover and reveal the molecular pathways and mechanisms of lncRNAs in plants.

Systematic screening and integrative crosscheck of lncRNAs would provide new insights to identify and functionally characterize potential key lncRNAs that are essential in plant response to and memory of various and repeated abiotic stresses. In addition, the conservation of their biological functions and metabolisms among different plant groups (such as Eudicotyledoneae and Monocotyledoneae, etc.) remains largely unclear. Therefore, large-scale assessments of plant lncRNA functionality and comparative analyses of lncRNA conservation across plant species will be powerful tools for identifying lncRNAs and studying their functions. GWAS-derived genomic analysis for functional candidate lncRNAs associated with abiotic stress in large populations could be employed to predict single-nucleotide polymorphisms and identify functional polymorphisms of lncRNAs. Furthermore, transposable elements, which play significant roles in evolution and are often found to be highly enriched in the upstream regions of lncRNAs and also regulate lncRNAs, might also be likely to regulate the adaption of plants to abiotic stresses, facilitating the evolution of land plants. Meanwhile, the conservation of lncRNAs might be underestimated, and new approaches capable of mapping lncRNA structure and interactions, as well as models capable of accurately capturing evolutionary constraints on lncRNA loci, need to be developed in order to discover the new biology of lncRNAs.

Growing evidence shows that in humans, the expression of lncRNAs is correlated with tumorigenesis, metastasis, and poor prognosis in many types of cancer, both in animal tests and clinical experiments. These findings indicate that many lncRNAs could be used as prognostic biomarkers and potential therapeutic targets [193,194]. Comparatively, the application of lncRNAs in plant breeding is still in its initial stages. Although lncRNAs mediate the regulation of plants in response to abiotic stresses in many species, their potential to be valuable genomic resources in plant molecular breeding or as indicators is yet to be confirmed. In addition, breeding strategies based on lncRNAs to optimize the balance between plant growth and abiotic stress conditions are required to be further developed [195]. To sum up, the potential applications of lncRNAs in plants are currently lacking. Much work remains to be completed in this area, due to the rapid evolution and multifaceted molecular functions of lncRNAs.

Taken together, the rapid and remarkable progress of lncRNAs in different plant species has significantly expanded our knowledge, but there are still many areas and papers that we have not included in this review. Despite the relevant results reported recently, the evolution, functions, mechanisms, and applications of plant lncRNAs under abiotic stresses remain to be understood. It will be a long journey, and more efforts are needed to fully understand their roles.

## Figures and Tables

**Table 1:** Statistics of lncRNAs in plant databases.

Database	Plant Species ^a^	Number of LncRNAs ^b^	Eudicotes	Monocots	Website	References
NONCODE v6.0	23	94,697	16 (69.6%)	5 (21.7%)	http://www.noncode.org/	[51]
PNRD	20	6152	14 (70%)	5 (25%)	http://structuralbiology.cau.edu.cn/PNRD/	[52]
AlnC	678	10,855,598	515 (76.0%)	100 (14.75%)	http://www.nipgr.ac.in/AlnC	[54]
PLncDB v2.0	80	1,246,372	52 (65.0%)	16 (20%)	http://plncdb.tobaccodb.org	[53]
LncPheDB	9	203,391	4 (44.4%)	5 (55.6%)	https://www.lncphedb.com/	[42]
CANTATAdb v2.0	39	239,631	20 (51.3%)	14 (35.9%)	http://cantata.amu.edu.pl, http://yeti.amu.edu.pl/CANTATA/	[55]
GreeNC v2.0	93	>495,000	53 (57.0%)	30 (32.3%)	http://greenc.sequentiabiotech.com/wiki2/Main_Page	[56]

^a^ Detailed plant species included in each database were listed in Supplemental Table S1. ^b^ Total numbers of lncRNA transcripts for all plant species.

**Table 2:** Representative lncRNAs found to be involved in plants responding to abiotic stresses.

Abiotic Function	Year	Plant Species	LncRNAs	References
Drought/dehydration	2023	*Glycine max*	*lncRNA77580*	[120]
	2022	*Oryza sativa* L.	*MSTRG.5679.8*; *MSTRG.19712.1*; *MSTRG.37152.2*	[75]
		*Arachis hypogaea* L.	*MSTRG.70535.2*; *MSTRG.86570.2*; *MSTRG.86570.1*; *MSTRG.100618.1*; *MSTRG.81214.2*; *MSTRG.30931.1*	[73]
		*Solanum lycopersicum*	*SlNCED1*; *SlAOC*; *SlLOX5*; *SlCWINV3-like*; *SlAgpL1*; *TomadPgps*; *SlMS1*	[77]
	2021	*Solanum tuberosum* L.	*StFLORE*	[121]
		*Oryza sativa* L.	*TCONS_00021861*	[62]
		*Gossypium hirsutum*	*GhDAN1*	[74]
	2020	*Brassica napus* L.	*XLOC_052298*; *XLOC_094954*; *XLOC_012868*	[68]
		*Zea mays*	*TCONS_00043110*; *TCONS_00077962*; *TCONS_00084669*; *TCONS-00105920*; *TCONS-00166326*; *TCONS-00060596*; *TCONS-00149876*; *TCONS-00177501*	[122]
	2019	*Oryza sativa* L.	*TCONS_00028567*	[123]
		*Manihot esculenta Crantz*	*TCONS_00097416*	[66]
		*Manihot esculenta Crantz*	*LNC_001148*; *LNC_000160*	[124]
		*Cleistogenes songorica*	*MSTRG.18766*; *MSTRG.22617.1*; *MSTRG.62661*	[72]
	2016	*Gossypium hirsutum*	*XLOC_063105*; *XLOC_115463*	[125]
Cold/heat stress	2022	*Manihot esculenta Crantz*	*CRIR1*	[126]
		*Picea glauca*	*MSTRG.33602.1*; *MSTRG.505746.1*; *MSTRG.1070680.1*	[127]
		*Arabidopsis thaliana*	*CIL1*	[128]
		*Oryza sativa* L.	*TCONS_00092993*; *TCONS_00043075*; *TCONS_00100154*	[129]
	2021	*Pyrus pyrifolia*	*TCONS_00028619*	[130]
		*Triticum aestivum* L.	*VAS*	[131]
		*Gossypium hirsutum*	*XH123*	[88]
		*Ziziphus jujuba Mill.*	*MSTRG.7381.6*; *MSTRG.20225.7*; *MSTRG.36975.1*; *MSTRG.25280.9*	[132]
		*Arabidopsis thaliana*	*XLOC_006026*	[133]
	2020	*Manihot esculenta Crantz*	*ncP12248*	[86]
		*Populus simonii*	*TCONS_00202587*; *TCONS_00260893*	[134]
		*Cucumis sativus* L.	*TCONS_00031790*; *TCONS_00014332*; *TCONS_00014717*; *TCONS_00005674*	[135]
		*Populus qiongdaoensis*	*lncHSP18.2*	[136]
	2019	*Triticum turgidum* L.	*Traes_2BS_7A04BF5D5.3*	[137]
		*Zea mays ssp. mays*	*MSTRG.73329*	[85]
		*Brassica rapa ssp.chinensis*	*TCONS_00048391*	[138]
	2018	*Arabidopsis thaliana*	*SVALKA*	[78]
	2014	*Arabidopsis thaliana*	*COOLAIR*	[139]
		*Arabidopsis thaliana*	*asHSFB2a*	[81]
	2011	*Arabidopsis thaliana*	*COLDAIR*	[140]
Salt/salinity	2023	*Medicago truncatula*	*MtCIR1*	[141]
	2023	*Glycine max*	*lncRNA77580*	[120]
	2022	*Populus Trichocarpa*	*Ptlinc-NAC72*	[142]
		*Zea mays*	*MSTRG.8888.1*	[143]
		*Glycine max*	*Gmax_MSTRG.35921.1*; *Gmax_MSTRG.18616.1*	[144]
	2021	*Gossypium hirsutum*	*lncRNA354*	[100]
	2020	*Lemna minor Linn.*	*TCONS_00033722*; *TCONS_00044328*; *TCONS_00059333*	[97]
		*Pistacia vera* L.	*lncRNA_PveLR32819*	[145]
	2019	*Gossypium* spp.	*lncRNA973*	[94]
		*Populus euphratica and P. alba var. pyramidalis*	*Pal_00132209*	[146]
	2018	*Gossypium hirsutum*	*lnc_388*; *lnc_973*; *lnc_253*	[147]
	2017	*Arabidopsis thaliana*	*DRIR*	[76]
	2015	*Medicago sativa L.*	*Medtr2g060880.1, Medtr3g071740.1, Medtr5g024020.2, Medtr4g098850.1, Medtr8g013680.1, and Medtr7g099800.1*	[148]
		*Medicago truncatula*	*TCONS_00046739*; *TCONS_00100258*; *TCONS_00118328*	[95]
	2009	*Arabidopsis thaliana*	*npcRNA536*	[149]
Heavy metal stress	2022	*Populus* L.	*PMAT*	[2]
	2021	*Populus tomentosa*	*MSTRG.22608.1*; *MSTRG.5634.1*	[150]
	2020	*Betula platyphylla*	*LncRNA2705.1*; *LncRNA11415.1*	[151]
	2018	*Oryza sativa* L.	*XLOC_086307*; *XLOC_058523*; *XLOC_104363*; *XLOC_059778*; *XLOC_122123*; *XLOC_125848*; *XLOC_098316*	[152]

## Data Availability

Not applicable.

## Supplementary Materials

The following supporting information can be downloaded at https://www.mdpi.com/article/10.3390/cells12050729/s1, Table S1: Plant species that included in each database.
