# Supplementary material for: Long Non-Coding RNAs of Plants in Response to Abiotic Stresses and Their Regulating Roles in Promoting Environmental Adaption
Source: Cells. 2023 Feb 24;12(5):729. doi: 10.3390/cells12050729 (PMC10001313; doi:10.3390/cells12050729)
Supplement: Supplementary file 1 [file cells-12-00729-s001.zip › cells-2181294-supplementary.pdf]

Supplemental Table S1. Plant species that included in each database

Database	Plant Species
**NONCODE v6.0**	*Arabidopsis thaliana*; *Brassica napus*; *Brassica rapa*; *Chenopodium quinoa*; *Chlamydomonas reinhardtii*; *Cucumber sativus*; *Glycine max*; *Gossypium raimondii*; *Musa acuminata*; *Malus domestica*; *Manihot esculenta*; *Medicago truncatula*; *Oryza rufipogon*; *Oryza sativa*; *Populus trichocarpa*; *Physcomitrella patens*; *Solanum lycopersicum*; *Solanum tuberosum*; *Triticum aestivum*; *Theobroma cacao*; *Trifolium pratense*; *Vitis vinifera*; *Zea mays*.
**PNRD**	*Arabidopsis lyrata*; *Arabidopsis thaliana*; *Brassica rapa*; *Cucumis sativus*; *Ganoderma lucidum*; *Gossypium barbadense*; *Hordeum vulgare subsp. Vulgare*; *Medicago sativa*; *Medicago truncatula*; *Nicotiana benthamiana*; *Nicotiana tabacum*; *Oryza sativa*; *Populus tomentosa*; *Populus tremula*; *Populus trichocarpa*; *Setaria italica*; *Solanum lycopersicum*; *Triticum aestivum*; *Vitis vinifera*; *Zea mays*.
**AlnC**	*Meliosma cuneifolia*; *Heliotropium greggii*; *Sassafras albidum*; *Boehmeria nivea*; *Papaver rhoeas*; *Galax urceolata*; *Linum leonii*; *Xerophyllum asphodeloides*; *Inula helenium*; *Cuscuta pentagona*; *Brugmansia sanguinea*; *Oenothera elata*; *Myodocarpus sp.*; *Terminalia neotaliala*; *Glycine soja*; *Atriplex hortensis*; *Ipomoea quamoclit*; *Humulus lupulus*; *Oenothera laciniata*; *Ilex vomitoria*; *Muntingia calabura*; *Acacia pycnantha*; *Scutellaria montana*; *Capnoides sempervirens*; *Phelline lucida*; *Cavendishia cuatrecasasii*; *Edgeworthia papyrifera*; *Thladiantha villosula*; *Sinojackia xylocarpa*; *Linum flavum*; *Ruellia brittoniana*; *Eucalyptus leucoxylon*; *Petiveria alliacea*; *Solenostemon scutellarioides*; *Tetrastigma obtectum*; *Kirkia wilmsii*; *Cinnamomum camphora*; *Zingiber officinale*; *Manilkara zapota*; *Zaleya pentandra*; *Cornus florida*; *Argemone mexicana*; *Cissus quadrangularis*; *Linum lewisii*; *Delosperma echinatum*; *Phytolacca americana*; *Hemerocallis spp.*; *Sorbus koehneana*; *Papaver somniferum*; *Senecio rowleyanus*; *Hypericum perforatum*; *Souroubea exauriculata*; *Atropa belladonna*; *Neurachne annularis*; *Psychotria ipecacuanha*; *Typha latifolia*; *Daenikera sp.*; *Chondropetalum tectorum*; *Gyrocarpus americanus*; *Philadelphus inodorus*; *Neurachne minor*; *Draba sachalinensis*; *Portulaca umbraticola*; *Brocchinia reducta*; *Posidonia australis*; *Mollugo spp.*; *Melia azedarach*; *Symphoricarpos sp.*; *Akebia trifoliata*; *Carthamus lanatus*; *Plumbago auriculata*; *Oenothera grandis*; *Bergenia sp.*; *Juncus inflexus*; *Oenothera nana*; *Ochna serrulata*; *Astilbe chinensis*; *Escallonia rubra*; *Escallonia sp. cv. Newport*; *Bacopa caroliniana*; *Hesperaloe parviflora*; *Lupinus polyphyllus*; *Malesherbia fasciculata*; *Polypremum procumbens*; *Triglochin maritima*; *Lophophora williamsii*; *Portulaca grandiflora*; *Convolvulus arvensis*; *Ulmus alata alata*; *Limnanthes douglasii*; *Houttuynia cordata*; *Cochlearia officinalis*; *Heliotropium texanum*; *Tellima breviflora*; *Basella alba*; *Blutaparon vermiculare*; *Mollugo nudicaulis*; *Simmondsia chinensis*; *Schlegelia parasitica*; *Heracleum lanatum*; *Betula pendula*; *Eleusine coracana*; *Cimicifuga racemosa*; *Portulaca oleracea*; *Moringa oleifera*; *Ardisia revoluta*; *Mitella pentandra*; *Alternanthera tenella*; *Calceolaria pinifolia*; *Plantago coronopus*; *Canella winterana*; *Tragopogon dubius*; *Erigeron canadensis*; *Ilex sp.*; *Cannabis sativa*; *Freycinetia multiflora*; *Geum quellyon*; *Eupomatia bennettii*; *Heliotropium tenellum*; *Mertensia paniculata*; *Solanum lasiophyllum*; *Solanum ptychanthum*; *Phycella aff. cyrtanthoides*; *Hamamelis virginiana*; *Vitex agnus-castus*; *Psychotria douarrei*; *Leontopodium alpinum*; *Zephyranthes treatiae*; *Kalanchoe crenato-diagremontiana*; *Asclepias curassavica*; *Oenothera biennis*; *Flaveria cronquistii*; *Sinningia tuberosa*; *Maesa lanceolata*; *Helenium autumnale*; *Tanacetum parthenium*; *Allionia incarnata*; *Sagittaria latifolia*; *Juglans nigra*; *Pycnanthemum tenuifolium*; *Oenothera picensis*; *Mirabilis jalapa*; *Batis maritima*;

Marrubium vulgare; *Amelanchier canadensis*; *Antirrhinum majus*; *Oenothera grandiflora*; *Oenothera filiformis*; *Gentiana acaulis*; *Polyscias fruticosa*; *Wrightia natalensis*; *Ficus religiosa*; *Schlegelia violacea*; *Dillenia indica*; *Heliotropium calcicola*; *Rhamnus japonica*; *Hedera helix*; *Anticharis glandulosa*; *Ehretia acuminata*; *Ipomoea pubescens*; *Aerva lanata*; *Borya sphaerocephala*; *Alternanthera brasiliana*; *Papaver setigerum*; *Oenothera berlandieri*; *Heuchera sanguinea*; *Flaveria angustifolia*; *Silybum marianum*; *Cleome violacea*; *Passiflora edulis*; *Nelumbo sp.*; *Calycanthus floridus*; *Conopholis americana*; *Saxifraga stolonifera*; *Boswellia sacra*; *Phormium tenax*; *Rosmarinus officinalis*; *Chamerion angustifolium*; *Valeriana officinalis*; *Syzygium paniculatum*; *Asparagus densiflorus*; *Phlox sp.*; *Greyia sutherlandii*; *Nepeta cataria*; *Tragopogon pratensis*; *Beta maritima*; *Alangium chinense*; *Garcinia oblongifolia*; *Stylidium adnatum*; *Oenothera rosea*; *Polygonum convolvulus*; *Daphniphyllum macropodum*; *Lavandula angustifolia*; *Austrobaileya scandens*; *Silene latifolia*; *Aerva persica*; *Oenothera speciosa*; *Thalictrum thalictroides*; *Verbena hastata*; *Portulaca suffrutescens*; *Gloriosa superba*; *Boerhavia burbidgeana*; *Byblis gigantea*; *Gleditsia triacanthos*; *Pogostemon sp.*; *Flaveria pringlei*; *Strychnos spinosa*; *Solanum dulcamara*; *Corokia cotoneaster*; *Aucuba japonica*; *Sarcobatus vermiculatus*; *Cypselea humifusa*; *Allium sativum*; *Salix eriocephala*; *Oxera neriifolia*; *Digitalis purpurea*; *Buddleja sp.*; *Grevillea robusta*; *Draba hispida*; *Anthemis tinctoria*; *Oenothera affinis*; *Draba aizoides*; *Berberidopsis beckleri*; *Pholisma arenarium*; *Aristida stricta*; *Aesculus pavia*; *Licania michauxii*; *Francoa appendiculata*; *Quercus shumardii*; *Gelsemium sempervirens*; *Astragalus membranaceus*; *Viburnum odoratissimum*; *Linum hirsutum*; *Nolina atopocarpa*; *Oenothera longituba*; *Loropetalum chinense*; *Utricularia sp.*; *Flaveria trinervia*; *Ancistrocladus tectorius*; *Tamarix chinensis*; *Paeonia lactiflora*; *Scaevola mossambicensis*; *Mollugo pentaphylla*; *Roridula gorgonias*; *Sabal bermudana*; *Flaveria vaginata*; *Flaveria pubescens*; *Serenoa repens*; *Akania lucens*; *Sesuvium portulacastrum*; *Deschampsia cespitosa*; *Rosa palustris*; *Yucca filamentosa*; *Oenothera elata hookeri*; *Heliotropium racemosum*; *Salix dasyclados*; *Crossopetalum rhacoma*; *Platycodon grandiflorus*; *Oenothera gaura*; *Quassia amara*; *Salix acutifolia*; *Myrica cerifera*; *Heliotropium filiforme*; *Flaveria brownii*; *Brassica nigra*; *Narcissus viridiflorus*; *Myriophyllum aquaticum*; *Buxus sempervirens*; *Brodiaea sierrae*; *Menyanthes trifoliata*; *Thymus vulgaris*; *Lobelia siphilitica*; *Ipomoea lobata*; *Bougainvillea spectabilis*; *Amaranthus palmeri*; *Apocynum androsaemifolium*; *Pinguicula caudata*; *Rhodophiala pratensis*; *Bauhinia tomentosa*; *Impatiens balsamifera*; *Holarrhena pubescens*; *Oxalis sp.*; *Hemerocallis sp.*; *Oenothera suffulta* ; *Melaleuca quinquenervia*; *Pereskia aculeata*; *Cicerbita plumieri*; *Maranta leuconeura*; *Datura metel*; *Koeberlinia spinosa*; *Symplocos tinctoria*; *Curcuma longa*; *Punica granatum*; *Masdevallia yuangensis*; *Bursera simaruba*; *Glycyrrhiza lepidota*; *Dipsacus asper*; *Aloe vera*; *Lindera benzoin*; *Oenothera clelandii*; *Allium commutatum*; *Linum macraei*; *Tragopogon castellanus*; *Tragopogon porrifolius*; *Salix viminalis*; *Heliconia sp.*; *Lathyrus sativus*; *Urginea maritima*; *Bixa orellana*; *Exacum affine*; *Peumus boldus*; *Chionanthus retusus*; *Kaliphora madagascariensis*; *Tribulus eichlerianus*; *Diospyros malabarica*; *Crassula perforata*; *Agave tequilana*; *Celtis occidentalis*; *Cyanella orchidiformis*; *Senna hebecarpa*; *Draba oligosperma*; *Atriplex rosea*; *Amaryllis belladonna*; *Haemaria discolor*; *Linum perenne*; *Salix purpurea*; *Portulaca amilis*; *Talinum sp.*; *Liquidambar styraciflua*; *Casuarina equisetifolia*; *Viola tricolor*; *Solanum xanthocarpum*; *Teucrium chamaedrys*; *Monotropa uniflora*; *Ruscus sp.*; *Orchidantha maxillarioides*; *Euphorbia mesembryanthemifolia*; *Sisyrinchium angustifolium*; *Alternanthera sessilis*; *Gomortega keule*; *Cleome gynandra*; *Boerhavia coccinea*; *Stackhousia spathulata*; *Pistia stratiotes*; *Allamanda*

cathartica; *Linum usitatissimum*; *Nicotiana sylvestris*; *Lactuca graminifolia*; *Phyla dulcis*; *Phytolacca bogotensis*; *Platanthera clavellata*; *Acorus americanus*; *Atriplex prostrata*; *Lomandra longifolia*; *Piper auritum*; *Sansevieria trifasciata*; *Griselinia littoralis*; *Smilax bona-nox*; *Pinguicula agnata*; *Astragalus propinquus*; *Tropaeolum peregrinum*; *Ligustrum sinense*; *Heliotropium mendocinum*; *Ipomoea lindheimeri*; *Sanchezia sp.*; *Prunus prostrata*; *Syzygium micranthum*; *Mammea americana*; *Ternstroemia gymnanthera*; *Ipomoea nil*; *Colchicum autumnale*; *Castanea crenata*; *Heliotropium karwinskyi*; *Heteropyxis natalensis*; *Pilostyles thurberi*; *Eschscholzia californica*; *Viola canadensis*; *Solanum sisymbriifolium*; *Hypecoum procumbens*; *Coriaria nepalensis*; *Neurachne tenuifolia*; *Ceratophyllum demersum*; *Nypa fruticans*; *Pittosporum sahnianum*; *Conyza canadensis*; *Cercidiphyllum japonicum*; *Flaveria palmeri*; *Kadsura heteroclita*; *Apios americana*; *Matricaria matricarioides*; *Hakea prostrata*; *Myristica fragrans*; *Peganum harmala*; *Begonia sp.*; *Dioscorea villosa*; *Ardisia humilis*; *Mollugo verticillata*; *Polygala lutea*; *Hydrocotyle umbellata*; *Hibbertia grossulariifolia*; *Schiedea membranacea*; *Hibiscus cannabinus*; *Eriospermum lancifolia*; *Linum bienne*; *Helonias bullata*; *Boykinia jamesii*; *Saururus cernuus*; *Sesuvium verrucosum*; *Ipomoea indica*; *Quillaja saponaria*; *Sarcandra glabra*; *Garcinia livingstonei*; *Lycium sp.*; *Deutzia scabra*; *Heliotropium convolvulaceum*; *Rehmannia glutinosa*; *Sideroxylon reclinatum*; *Aristolochia elegans*; *Ricinus communis*; *Anisacanthus quadrifidus*; *Psychotria marginata*; *Glycyrrhiza glabra*; *Phellodendron amurense*; *Prunella vulgaris*; *Orobanche fasciculata*; *Cistus inflatus*; *Chlorogalum pomeridianum*; *Rhododendron scopulorum*; *Typha angustifolia*; *Agapanthus africanus*; *Lantana camara*; *Uvaria microcarpa*; *Plantago virginica*; *Jacquinia sp.*; *Staphylea trifolia*; *Agastache rugosa*; *Daphne giraldii*; *Cyperus papyrus*; *Euphorbia pekinensis*; *Nymphaea sp.*; *Helwingia japonica*; *Opuntia polyacantha*; *Actinidia chinensis*; *Flaveria bidentis*; *Saruma henryi*; *Rauvolfia tetraphylla*; *Cocos nucifera*; *Ailanthus altissima*; *Cotoneaster transcaucasicus*; *Griselinia racemosa*; *Wikstroemia indica*; *Kigelia africana*; *Phoradendron serotinum*; *Trianthema portulacastrum*; *Sanguisorba minor*; *Xerophyta villosa*; *Sambucus canadensis*; *Polanisia trachysperma*; *Ipomoea hederacea*; *Euptelea pleiosperma*; *Dichroa febrifuga*; *Aextoxicon punctatum*; *Flaveria kochiana*; *Gymnocladus dioicus*; *Pyrenacantha malvifolia*; *Elaeagnus pungens*; *Uniola paniculata*; *Maianthemum sp.*; *Cyanastrum cordifolium*; *Lagerstroemia indica*; *Cercis canadensis*; *Copaifera officinalis*; *Avena fatua*; *Wisteria floribunda*; *Mollugo cerviana*; *Illicium parviflorum*; *Cymbopogon nardus*; *Erythroxylum coca*; *Nolina bigelovii*; *Papaver bracteatum*; *Santalum acuminatum*; *Oxera pulchella*; *Salvadora sp.*; *Physena madagascariensis*; *Drypetes deplanchei*; *Saintpaulia ionantha*; *Polycarpaea repens*; *Salix fargesii*; *Pittosporum resiniferum*; *Xeronema callistemon*; *Pennantia corymbosa*; *Ipomoea purpurea*; *Sarcodes sanguinea*; *Hilleria latifolia*; *Dianthus caryophyllus*; *Celsia arcturus*; *Hakea drupacea*; *Talbotia elegans*; *Passiflora caerulea*; *Oenothera serrulata*; *Saponaria officinalis*; *Tiarella polyphylla*; *Cladrastis lutea*; *Chenopodium quinoa*; *Lennoa madreporoides*; *Panicum miliaceum*; *Dryas octopetala*; *Balanophora fungosa*; *Codariocalyx motorius*; *Drimia altissima*; *Fagus sylvatica*; *Tetrazygia bicolor*; *Trochodendron aralioides*; *Reseda odorata*; *Physocarpus opulifolius*; *Ilex paraguariensis*; *Ribes aff. giraldii*; *Tetrastigma voinierianum*; *Eucommia ulmoides*; *Melissa officinalis*; *Peliosanthes minor*; *Salix sachalinensis*; *Solidago canadensis*; *Vanilla planifolia*; *Elaeocarpus sylvestris*; *Cunonia capensis*; *Spergularia media*; *Nothofagus obliqua*; *Kerria japonica*; *Mansoa alliacea*; *Portulaca cryptopetala*; *Aphanopetalum resinosum*; *Olea europaea*; *Platyspermation crassifolium*; *Angelica archangelica*; *Lupinus angustifolius*; *Ochna mossambicensis*; *Linum strictum*; *Canna sp.*; *Arabis*

	alpina; *Gyrostemon ramulosus*; *Xanthium strumarium*; *Ajuga reptans*; *Ceratocapnos vesicaria*; *Larrea tridentata*; *Forestiera segregata*; *Lycopersicon cheesmanii*; *Oresitrophe rupifraga*; *Citrus x paradisi*; *Morinda citrifolia*; *Paulownia fargesii*; *Strelitzia reginae*; *Catharanthus roseus*; *Anemone pulsatilla*; *Cleome viscosa*; *Portulaca molokiniensis*; *Amborella trichopoda*; *Epifagus virginiana*; *Tabebuia umbellata*; *Azadirachta indica*; *Draba magellanica*; *Itea virginica*; *Flaveria sonorensis*; *Curtisia dentata*; *Castanea pumila*; *Disporopsis pernyi*; *Stemona tuberosa*; *Malus baccata var. jackii*; *Acer negundo*; *Hydrastis canadensis*; *Dendropemon caribaeus*; *Gleditsia sinensis*; *Gompholobium polymorphum*; *Sinapis alba*; *Bischofia javanica*; *Neurachne lanigera*; *Caiophora chuquitensis*; *Goodyera pubescens*; *Nyssa ogeche*; *Croton tiglium*; *Ludovia sp.*; *Carya glabra*; *Linum tenuifolium*; *Stachyurus praecox*; *Cassytha filiformis*; *Illicium floridanum*; *Laurelia sempervirens*; *Litchi chinensis*; *Lepidosperma gibsonii*; *Magnolia grandiflora*; *Thyridolepis multiculmis*; *Strobilanthes dyeriana*; *Centella asiatica*; *Podophyllum peltatum*; *Kochia scoparia*; *Alternanthera caracasana*; *Micromeria fruticosa*; *Persea borbonia*; *Urtica dioica*; *Drimys winteri*; *Amaranthus retroflexus*; *Schizolaena sp.*; *Limonium spectabile*; *Idiospermum australiense*; *Frankenia laevis*; *Galium boreale*; *Nepenthes alata*; *Synsepalum dulcificum*; *Johnsonia pubescens*; *Nuphar advena*; *Lindenbergia philippensis*; *Rhamnus caroliniana*; *Phlox drummondii*; *Tapiscia sinensis*; *Medinilla magnifica*; *Joinvillea ascendens*; *Ledum palustre*; *Dombeya burgessiae*; *Ascarina rubricaulis*; *Thyridolepis mitchelliana*; *Cercocarpus ledifolius*; *Maianthemum canadense*; *Exocarpos cupressiformis*; *Sanguinaria canadensis*; *Ximenia americana*; *Poliomintha bustamanta*; *Gunnera manicata*; *Chelidonium majus*; *Manihot grahamii*; *Camptotheca acuminata*; *Desmanthus illinoensis*; *Mapania palustris*; *Galphimia gracilis*; *Michelia maudiae*; *Aster tataricus*; *Buddleja lindleyana*; *Amaranthus tricolor*; *Peperomia fraseri*; *Paraneurachne muelleri*; *Morus nigra*; *Verbascum sp.*; *Drakaea elastica*; *Asclepias syriaca*; *Yucca brevifolia*; *Phyllanthus sp.*; *Geranium maculatum*; *Nandina domestica*; *Oenothera rhombipetala*; *Curculigo sp.*; *Peltoboykinia watanabei*; *Plantago maritima*; *Typhonium blumei*; *Microtea debilis*; *Microstegium vimineum*; *Phacelia campanularia*; *Antirrhinum braun-blanquetii*; *Fouquieria macdougalii*; *Rhus radicans*; *Triodia aff. bynoei*; *Cyrilla racemiflora*; *Annona muricata*; *Cephalotus follicularis*; *Chrysobalanus icaco*; *Acacia argyrophylla*; *Neurachne alopecuroidea*; *Hydrangea quercifolia*; *Corydalis linstowiana*; *Ziziphus jujuba*; *Krameria lanceolata*; *Rhodiola rosea*; *Traubia modesta*; *Neurachne munroi*; *Aruncus dioicus*; *Uncarina grandidieri*; *Hoheria angustifolia*; *Xanthocercis zambesiaca*; *Rhizophora mangle*; *Anemone hupehensis*.
**PLncDB v2.0**	*Actinidia chinensis*; *Amborella trichopoda*; *Ananas comosus*; *Aquilegia coerulea*; *Arabidopsis lyrata*; *Arabidopsis thaliana*; *Arachis ipaensis*; *Brachypodium distachyon*; *Brassica napus*; *Brassica rapa*; *Capsella grandiflora*; *Capsella rubella*; *Capsicum annuum*; *Carica papaya*; *Chlamydomonas reinhardtii*; *Cicer arietinum*; *Citrus clementina*; *Citrus maxima*; *Citrus sinensis*; *Coccomyxa subellipsoidea*; *Coffea arabica*; *Cucumis sativus*; *Daucus carota*; *Dunaliella salina*; *Durio zibethinus*; *Elaeis guineensis*; *Erythranthe guttata*; *Eucalyptus grandis*; *Eutrema salsugineum*; *Fragaria vesca*; *Ganoderma lucidum*; *Glycine max*; *Gossypium barbadense*; *Gossypium raimondii* ; *Hordeum vulgare*; *Jatropha curcas*; *Lactuca sativa*; *Linum usitatissimum*; *Lotus japonicus*; *Lupinus angustifolius*; *Malus domestica*; *Manihot esculenta* ; *Medicago truncatula*; *Micromonas commoda*; *Micromonas pusilla*; *Musa acuminata*; *Nicotiana benthamiana*; *Nicotiana tabacum*; *Olea europaea*; *Oryza brachyantha*; *Oryza sativa*; *Ostreococcus lucimarinus*; *Panax ginseng*; *Panicum hallii* ; *Phaseolus vulgaris*; *Phoenix dactylifera*; *Physcomitrella patens*; *Pisum sativum*; *Populus tremula*; *Populus trichocarpa*; *Prunus*

	persica; *Ricinus communis*; *Salvia miltiorrhiza*; *Selaginella moellendorffii*; *Setaria italica*; *Setaria viridis*; *Solanum lycopersicum*; *Solanum melongena*; *Solanum pimpinellifolium*; *Solanum tuberosum*; *Sorghum bicolor*; *Spirodela polyrhiza*; *Theobroma cacao*; *Trifolium pratense*; *Triticum aestivum*; *Vigna radiata*; *Vitis vinifera*; *Volvox carteri*; *Zea mays*; *Zostera marina*.
**LncPheDB**	*Zea mays*; *Gossypium barbadense*; *Triticum aestivum*; *Lycopersicon esculentum*; *Oryza sativa*; *Hordeum vulgare*; *Sorghum Bicolor*; *Glycine max*; *Cucumis sativus*.
**CANTATAdb v2.0**	*Amborella trichopoda*; *Ananas comosus*; *Arabidopsis lyrate*; *Arabidopsis thaliana*; *Brachypodium distachyon*; *Brassica napus*; *Brassica oleracea*; *Brassica rapa*; *Chenopodium quinoa*; *Chlamydomonas reinhardtil*; *Chondrus crispus*; *Corchorus capsularis*; *Cucumis sativus*; *Galdieria sulphuraria*; *Glycine max*; *Hordeum vulgare*; *Leersia perrieri*; *Malus domestica*; *Manihot esculenta*; *Medicago truncatula*; *Musa acuminata*; *Oryza barthi*; *Oryza brachyantha*; *Oryza nivara*; *Oryza punctata*; *Oryza rufipogon*; *Oryza sativa*; *Physcomitrella patens*; *Populus trichocarpa*; *Prunus persica*; *Selaginella moellendorffi*; *Setaria italica*; *Solanum lycopersicum*; *Solanum tuberosum*; *Sorghum bicolor*; *Theobroma cacao*; *Trifolium pratense*; *Vitis vinifera*; *Zea mays*.
**GreeNC v2.0**	*Actinidia chinensis*; *Aegilops tauschii*; *Amaranthus hypocondriacus*; *Aquilegia coerulea*; *Arabidopsis halleri*; *Arabidopsis lyrata*; *Arabidopsis thaliana*; *Arabis alpina*; *Beta vulgaris*; *Boechera stricta*; *Brachypodium stacei*; *Brassica napus*; *Brassica rapa*; *Camelina sativa*; *Capsella rubella*; *Capsicum annuum*; *Chara braunii*; *Chenopodium quinoa*; *Chondrus crispus*; *Chromochloris zofingiensis*; *Cicer arietinum*; *Cinnamomum kanehirae*; *Citrullus lanatus*; *Coffea canephora*; *Corchorus capsularis*; *Cucumis melo*; *Cucumis sativus*; *Cyanidioschyzon merolae*; *Cynara cardunculus*; *Dioscorea alata*; *Eragrostis curvula*; *Eragrostis tef*; *Glycine max*; *Gossypium barbadense*; *Gossypium darwinii*; *Gossypium hirsutum*; *Gossypium mustelinum*; *Gossypium raimondii*; *Gossypium tomentosum*; *Helianthus annuus*; *Hordeum vulgare*; *Juglans regia*; *Kalanchoe fedtschenkoi*; *Lactuca sativa*; *Leersia perrieri*; *Lupinus albus*; *Lupinus angustifolius*; *Manihot esculenta*; *Marchantia polymorpha*; *Medicago truncatula*; *Musa acuminata*; *Nicotiana attenuata*; *Nymphaea colorata*; *Olea europaea*; *Oropetium thomaeum*; *Oryza barthii*; *Oryza brachyantha*; *Oryza glaberrima*; *Oryza glumipatula*; *Oryza longistaminata*; *Oryza meridionalis*; *Oryza nivara*; *Oryza punctata*; *Oryza rufipogon*; *Oryza sativa*; *Panicum hallii*; *Panicum virgatum*; *Papaver somniferum*; *Pistacia vera*; *Poncirus trifoliata*; *Porphyra umbilicalis*; *Prunus avium*; *Prunus dulcis*; *Quercus lobata*; *Rosa chinensis*; *Saccharum spontaneum*; *Salix purpurea*; *Sesamum indicum*; *Setaria viridis*; *Solanum lycopersicum*; *Solanum tuberosum*; *Sorghum bicolor*; *Trifolium pratense*; *Triticum aestivum*; *Triticum dicoccoides*; *Triticum spelta*; *Triticum turgidum*; *Triticum urartu*; *Vigna angularis*; *Vigna radiata*; *Vigna unguiculata*; *Vitis vinifera*; *Zea mays*.
